# Focus on chest trauma: implications from clinical and experimental studies

**DOI:** 10.1007/s00068-020-01310-7

**Published:** 2020-02-17

**Authors:** Klemens Horst, Frank Hildebrand

**Affiliations:** Department of Trauma and Reconstructive Surgery, Uniklinik RWTH Aachen, Aachen, Germany

Chest trauma still represents one of the most frequent and devastating injuries after polytrauma. Besides the direct effects of the traumatic impact itself, lung integrity and function are also indirectly endangered by the systemic release of inflammatory mediators due to additional injuries of other body regions. Significant efforts have to be made to better understand the underlying pathomechanisms to improve diagnostic and therapeutic strategies. This issue of the *European Journal of Trauma and Emergency Surgery* therefore focuses on recent aspects of chest trauma based on data from clinical and experimental studies, which underlines the relevance of bidirectional translational research.

The relevance of complications after chest trauma was a focus of the clinical studies of this issue. In a retrospective analysis, Huang et al. observed over an 8-year period [1] that almost 50% of the 10,362 trauma patients included in the study presented with chest trauma. Overall, thoracic injuries represented a risk factor for the development of adult respiratory distress syndrome (ARDS). However, ARDS-associated mortality was tenfold higher in patients without chest trauma. In these patients, ventilator-associated pneumonia (VAP) was the only preventable and treatable risk factor. In another clinical study, Hofman et al. analyzed the relevance of chest trauma and traumatic brain injury (TBI) in severely injured patients (ISS ≥ 16) [2]. In their retrospective analysis, chest trauma was an independent predictor of pneumonia. Pneumonia in turn represented the strongest independent predictor of in-hospital mortality, followed by combined chest trauma and TBI, TBI alone, and the duration of ventilation. The authors concluded that chest trauma seems to represent a direct risk factor, whereas TBI acts more as an indirect risk factor via a prolonged duration of ventilation. Duration of ventilation is by itself an independent predictor of pneumonia after trauma. Both studies, therefore, concluded that innovative prevention and treatment protocols for pneumonia have to be developed in future studies.

Due to a significant heterogeneity of the trauma population, experimental studies are of upmost importance to investigate the underlying pathomechanisms of posttraumatic complications and to establish new strategies. A model was introduced by Stormann et al., who investigated contributing factors for the development of acute lung injury (ALI) in a murine double hit model [3]. To induce ALI, the authors induced either a double hit with blunt chest trauma and cecal ligation and puncture (CLP) or chest trauma alone. It was observed that lung injury alone was associated with moderate inflammatory changes and quick recovery while the double hit resulted in a significant activation of the inflammatory response and unfavorable outcomes. This model will help to discriminate the influence of direct (chest trauma) and indirect (e.g. systemic inflammatory response) in future experimental studies. The importance of factors potentially associated with indirect lung damage was further investigated by Stormann et al. in a porcine long-term trauma model [4]. In their study, the authors focused on the effects of fracture fixation on remote organ damage in an isolated femur fracture model and an established polytrauma (PT) model that included femur fracture, chest trauma, liver injury, and hemorrhagic shock. Over the 72-h posttraumatic observation period which occurred under clinically realistic conditions (fracture fixation, resuscitation, intensive care treatment with mechanical ventilation), the authors found an increase of pro-inflammatory mediators and histological damage in the lung after MT and PT. Polytrauma was associated with a substantial increase in the intensity of the inflammatory response and organ damage. These findings might aid in understanding the pathophysiological background for the impact of surgical treatment strategies on the posttraumatic course. Furthermore, this large animal models seems suitable to investigate the relevance of new diagnostic and therapeutic approaches.

These innovations in diagnosis and therapy are essential to reliably prevent the patient from further harm and to improve posttraumatic outcomes. In this context, reliable assessment of the patient status is also necessary. Caspers et al. conducted a clinical study focusing on the role of microparticles (MP) after severe trauma [5]. The authors found a characteristic MP phenotype pattern which was associated with specific, trauma-induced alterations such as coagulopathy and impaired haemostatic capacity. The authors urge further investigations to elucidate the emerging role of microvesicles (MV) for diagnosis and potential treatment options of polytrauma patients. Tanriverdi et al. investigated possible therapeutic options in a rat trauma model [6]. The authors applied mesenchymal stem cells (MSC) after severe trauma including liver and bone injury. MSC administration was associated with an attenuation of local inflammation as well as improved histological findings. The authors concluded that MSC infusion might be beneficial for posttraumatic tissue healing. However, inclusion criteria and standard protocols in humans are not yet defined and have to be established before clinical trials are performed.

In conclusion, the articles presented here reflect the current problems in patients with chest trauma and offer promising experimental models to study the pathomechanisms associated with this trauma entity. Furthermore, interesting aspects regarding future directions in diagnostic and therapeutic options are illustrated.

We hope you enjoy reading our selections of topics around the importance of chest trauma in the clinical and experimental settings.
